# Aloe-Emodin Derivative, an Anthraquinone Compound, Attenuates Pyroptosis by Targeting NLRP3 Inflammasome in Diabetic Cardiomyopathy

**DOI:** 10.3390/ph16091275

**Published:** 2023-09-08

**Authors:** Yingying Hu, Shuqian Zhang, Han Lou, Monayo Seth Mikaye, Run Xu, Ziyu Meng, Menghan Du, Pingping Tang, Zhouxiu Chen, Yongchao Chen, Xin Liu, Zhimin Du, Yong Zhang

**Affiliations:** 1 Department of Pharmacology (The State-Province Key Laboratories of Biomedicine-Pharmaceutics of China, Key Laboratory of Cardiovascular Research, Ministry of Education), College of Pharmacy, Harbin Medical University, Harbin 150086, China; 2 Research Unit of Noninfectious Chronic Diseases in Frigid Zone, Chinese Academy of Medical Sciences, 2019RU070, Harbin 150081, China; 3 Institute of Clinical Pharmacology, The Second Affliated Hospital of Harbin Medical University (University Key Laboratory of Drug Research, Heilongjiang Province), Harbin 150086, China; 4 Department of Clinical Pharmacology College of Pharmacy, Harbin Medical University, Harbin 150081, China; 5 State Key Laboratory of Quality Research in Chinese Medicines, Macau University of Science and Technology, Macau 999078, China; 6 Institute of Metabolic Disease, Heilongjiang Academy of Medical Science, Harbin 150086, China

**Keywords:** aloe-emodin derivative, diabetic cardiomyopathy, pyroptosis, NLRP3 inflammasome

## Abstract

Diabetic cardiomyopathy (DCM) is widely recognized as a major contributing factor to the development of heart failure in patients with diabetes. Previous studies have demonstrated the potential benefits of traditional herbal medicine for alleviating the symptoms of cardiomyopathy. We have chemically designed and synthesized a novel compound called aloe-emodin derivative (AED), which belongs to the aloe-emodin (AE) family of compounds. AED was formed by covalent binding of monomethyl succinate to the anthraquinone mother nucleus of AE using chemical synthesis techniques. The purpose of this study was to investigate the effects and mechanisms of AED in treating DCM. We induced type 2 diabetes in Sprague–Dawley (SD) rats by administering a high-fat diet and streptozotocin (STZ) injections. The rats were randomly divided into six groups: control, DCM, AED low concentration (50 mg/kg/day), AED high concentration (100 mg/kg/day), AE (100 mg/kg/day), and positive control (glyburide, 2 mg/kg/day) groups. There were eight rats in each group. The rats that attained fasting blood glucose of ˃16.7 mmol/L were considered successful models. We observed significant improvements in cardiac function in the DCM rats with both AED and AE following four weeks of intragastric treatment. However, AED had a more pronounced therapeutic effect on DCM compared to AE. AED exhibited an inhibitory effect on the inflammatory response in the hearts of DCM rats and high-glucose-treated H9C2 cells by suppressing the pyroptosis pathway mediated by the nucleotide-binding oligomerization domain (NOD)-like receptor pyrin domain 3 (NLRP3) inflammasome. The Kyoto Encyclopedia of Genes and Genomes (KEGG) pathway analysis of differentially expressed genes showed a significant enrichment in the NOD-like receptor signaling pathway compared to the high-glucose group. Furthermore, overexpression of NLRP3 effectively reversed the anti-pyroptosis effects of AED in high-glucose-treated H9C2 cells. This study is the first to demonstrate that AED possesses the ability to inhibit myocardial pyroptosis in DCM. Targeting the pyroptosis pathway mediated by the NLRP3 inflammasome could provide a promising therapeutic strategy to enhance our understanding and treatment of DCM.

## 1. Introduction

Diabetic cardiomyopathy (DCM) is characterized as an abnormality in the myocardial structure and function in patients with diabetes, which cannot be attributed to hypertension, coronary artery disease, congenital heart disease, or other cardiac disorders [1]. DCM has the potential to progress to heart failure and sudden death. Recent reports indicate that it is the leading cause of mortality in patients suffering from diabetes [2]. According to a population-based prevalence study conducted in 2014, it was estimated that patients with DCM have a cumulative probability of 31% for developing heart failure or death [3]. The growing morbidity and mortality of DCM patients necessitates the development of effective drugs to curb this malady. To date, the exact mechanisms underlying the development of DCM remain to be fully established, largely due to its multifactorial etiology and complex nature. However, several molecular mechanisms involving metabolic disorders, such as hyperglycemia, lipotoxicity, and insulin resistance, have been proposed to contribute to cardiac inflammation, ultimately leading to the onset and progression of DCM [4,5].

Pyroptosis, a form of programmed cell death triggered by inflammation, has been implicated in the development of DCM [6,7]. Hyperglycemia plays a role in stimulating and activating nucleotide-binding oligomerization domain (NOD)-like receptor pyrin domain 3 (NLRP3) and adaptor protein apoptosis-associated speck-like protein (ASC) [8]. This subsequently leads to the activation of caspase-1 and gasdermin D (GSDMD), resulting in the induction of pyroptosis and the release of interleukins, such as interleukin-1β (IL-1β) and interleukin-18 (IL-18) [9,10]. These interleukins have previously been reported as instigators of various pathological processes that contribute to the development of DCM [11]. Consequently, drugs targeting the pyroptosis pathway may be effective in preventing or treating DCM. 

Studies on traditional herbal medicine have shown beneficial effects on DCM [12,13,14,15]. Aloe emodin (AE) (1,8-dihydroxy-3-hydroxyl-methylanthraquinone) is an anthraquinone derivative isolated as an exudate from the aloe plant. In recent years, it has been widely implicated in neoplasm through a dependent apoptotic pathway [16,17]. Additionally, the anti-inflammatory properties of AE have been well documented. Alshwati et al. reported that AE provided protective effects against high-glucose-induced glucotoxicity on β-cells, resulting in decreased levels of pro-inflammatory cytokines, such as IL-1β [18]. Zhang et al. revealed a new protective mechanism of AE involving the inhibition of NLRP3 inflammasome activation and a decrease in high-mobility group box1 (HMGB1) release by promoting NLRP3 ubiquitination [19]. More recently, Chen et al. established that AE could alleviate cardiac inflammation induced by high-fat diet/palmitic acid through the inhibition of the toll-like receptor 4 (TLR4)/nuclear factor-κB (NF-κB) signaling pathway [20]. 

**Figure 1:**
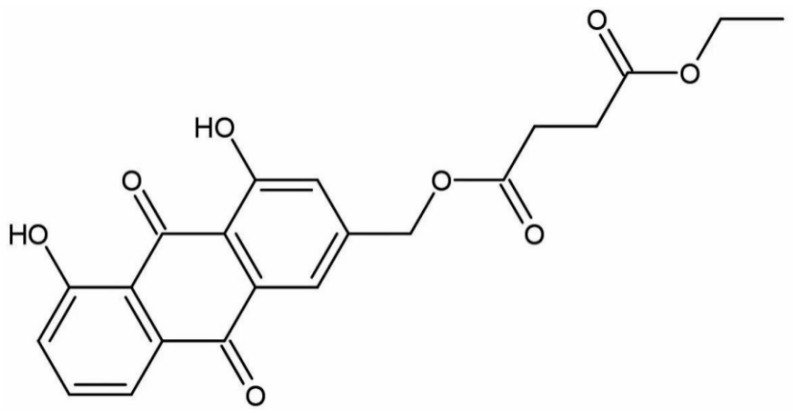
Structure of aloe-emodin derivative.

In this study, we chemically designed and synthesized a novel anthraquinone compound belonging to the AE family. We created a novel compound known as aloe-emodin derivative (AED) (1,8-dihydroxy-9,10-anthraquinone-3-diethyl succinate) by covalent binding of monomethyl succinate to the anthraquinone mother nucleus of AE using chemical synthesis techniques (Figure 1) [21,22]. The study illustrates the potential of AED to ameliorate hyperglycemia in a type 2 diabetes rat model, as well as in high-glucose-induced glucotoxicity in H9C2 cells. In addition, we evaluated the pharmacological efficacy of AED in both in vivo and in vitro settings, with a focus on its impact on pyroptosis as an underlying molecular mechanism.

## 2. Results

### 2.1. AED Attenuates Cardiac Dysfunction in Type 2 Diabetic Rats

**Figure 2:**
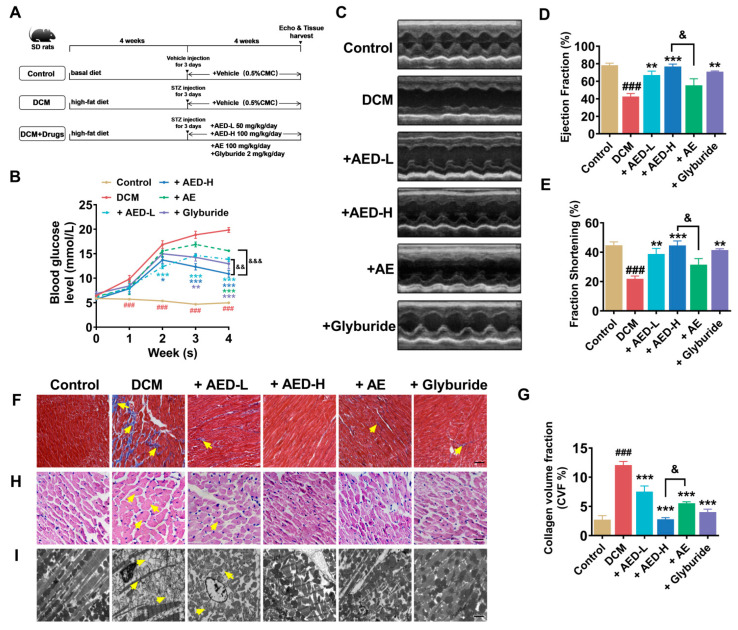
AED and AE improve cardiac function in DCM rats. (**A**) The flowchart of different treatments to build the rat models. (**B**) Fasting blood glucose measured after 4 weeks of STZ injection in the DCM and drug-treated groups. *n* = 5. (**C**) Representative images of echocardiography. (**D**) Ejection fraction (EF) and (**E**) fractional shortening (FS) measurements before the rats were sacrificed. *n* = 4. (**F**) Masson’s trichome staining (×200) was used to measure collagen deposition in the heart. The fibrotic area was stained blue. Scale bar: 200 µm. (**G**) Quantitative analysis of fibrosis, as revealed by Masson’s trichrome staining. The area of fibrosis was quantified using ImageJ software and expressed as the percentage of tissue area. *n* = 5. (**H**) H&E staining (×400) for histopathological estimations. Scale bar: 100 µm. *n* = 5. (**I**) Transmission electron microscopy used to show the ultrastructural changes in cardiac tissue. Scale bar: 2 µm. *n* = 3. The yellow arrows indicate the histopathological and electron microscopic changes in the different groups. * *p* < 0.05, ** *p* < 0.01, *** *p* < 0.001 vs. DCM; ^###^ *p* < 0.001 vs. control; ^&^ p *<* 0.05, ^&&^ *p* < 0.01, ^&&&^ *p* < 0.001 vs. +AED-H. The data are represented as the mean ± SEM. DCM, diabetic cardiomyopathy; AED-L, aloe-emodin derivative low concentration; AED-H, aloe-emodin derivative high concentration; AE, aloe emodin.

We induced DCM by administering a high-fat diet in combination with streptozotocin (STZ) injections to Sprague–Dawley (SD) rats. The experimental procedures and timeline are presented in Figure 2A. The DCM rats exhibited significantly elevated levels of fasting blood glucose, as shown in Figure 2B, demonstrating the successful establishment of the type 2 diabetes model. Plasma glucose levels remained within a normal range in the control animals, whereas these parameters were significantly improved in drug-treated groups. The above results indicate that both AED and AE have the capability to ameliorate hyperglycemia in type 2 diabetes. To determine whether AED and AE could improve cardiac function in diabetic rats, echocardiography was performed on all six groups four weeks after STZ injection. Notably, DCM rats exhibited a remarkable decline in left-ventricular ejection fraction (EF) and left-ventricular fraction shortening (FS), as shown in Figure 2C–E. These measurements were significantly ameliorated in the AE and AED-treated groups. Notably, AED demonstrated more pronounced effects compared to AE and exhibited similar efficacy as glyburide. Furthermore, histopathological analysis, including Masson’s trichrome staining and hematoxylin and eosin (H&E) staining of myocardial tissue, revealed structural abnormalities, such as interstitial fibrotic areas, increased myocardial cell size, and increased cross-sectional areas, indicating cardiac remodeling and hypertrophy, which are significant features of DCM (Figure 2F–H). However, these histological features were ameliorated in the drug-treated groups. Furthermore, transmission electron microscopy analysis showed that, in comparison to the DCM group, the AED and AE treatment groups exhibited improved ultrastructural changes, including improved myofilament breakage, reduced mitochondrial swelling and damage (Figure 2I). These findings indicate that both AED and AE have the capability to improve cardiac function in DCM rats. It is worth mentioning that AED exhibited higher potency compared to AE at the same dose, indicating its potential as a more effective treatment option.

### 2.2. AED Downregulates Pyroptosis Related Genes in the Hearts of DCM Rats

**Figure 3:**
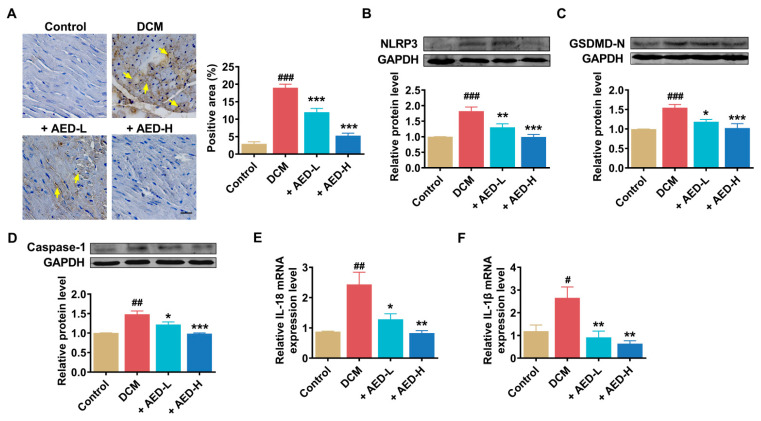
Pyroptotic gene analysis in DCM rats. (**A**) Immunohistochemical staining for myocardial NLRP3 expression. Brown staining indicates the cells with positive expression. The yellow arrows indicate the obvious changes of NLRPs expression in the different groups. Scale bar: 10 μm. Statistical results of the NLRP3-positive area was quantified using ImageJ software and expressed as the percentage of tissue area. *n* = 5. (**B**–**D**) The protein expression levels of NLRP3, GSDMD-N, and caspase-1 were examined by Western blot analysis. (**E**,**F**) The mRNA levels of IL-18 and IL-1β were examined by quantitative real-time PCR. *n* = 3–5. GAPDH was used as an internal control. * *p* < 0.05, ** *p* < 0.01, *** *p* < 0.001 vs. DCM; ^#^ *p* < 0.05, ^##^ *p* < 0.01, ^###^ *p* < 0.001 vs. control. The data are represented as the mean ± SEM. DCM, diabetic cardiomyopathy; AED-L, aloe-emodin derivative low concentration; AED-H, aloe-emodin derivative high concentration.

Several studies have highlighted the important roles of NLRP3-mediated pyroptosis in DCM. To explore whether AED exhibits its anti-DCM effects through the regulation of pyroptosis, we tested NLRP3-mediated pyroptosis in heart tissue from different groups of rats. Immunohistochemical analysis showed a remarkable increase in the expression of NLRP3 in the DCM group in comparison with the control group (Figure 3A). However, compared to the DCM group, the positive staining was decreased in AED-treated groups, particularly in the higher-concentration group. Additionally, these histological changes were further supported by Western blot analysis of NLPR3 protein (Figure 3B). The protein levels of GSDMD-N and caspase-1 were significantly increased in the DCM group, whereas AED treatment resulted in a decrease in the expression of these proteins (Figure 3C,D). The mRNA levels of genes associated with pyroptosis, including IL-18 and IL-1β, were considerably elevated in the DCM group, but AED treatment significantly restored these effects (Figure 3E,F). Collectively, these results provide strong evidence that AED treatment has a beneficial effect on pyroptosis induced by DCM.

### 2.3. Dose-Dependent High-Glucose-Induced Pyroptosis in H9C2 Cells

**Figure 4:**
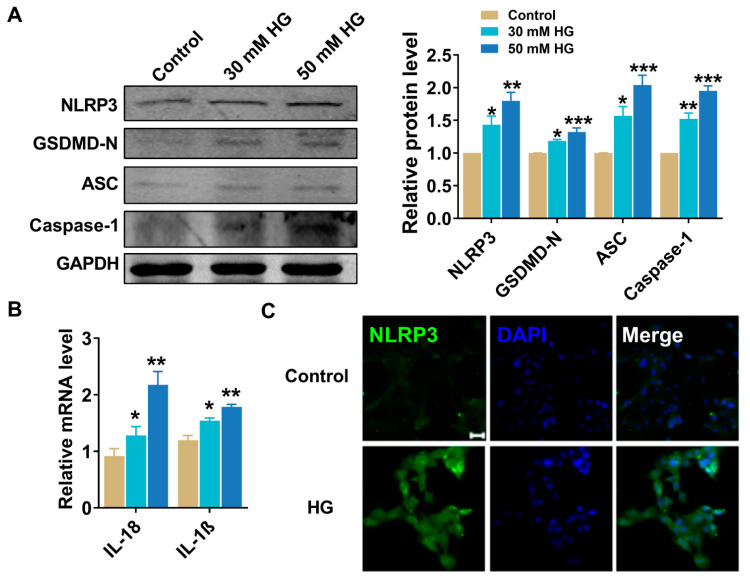
High glucose promotes pyroptosis in H9C2 cells. (**A**,**B**) Relative protein levels of NLRP3, GSDMD-N, ASC, and caspase-1 treated with different concentrations of high glucose (30 mM and 50 mM). *n* = 3–5. (**B**) Relative mRNA levels of IL-18 and IL-1β treated with different concentrations of high glucose (30 mM and 50 mM). *n* = 3–5. (**C**) Immunofluorescence results (×200) indicating the expression of NLRP3 (comparison between control and high-glucose-treated H9C2 cells). Scale bar: 20 μm. Blue: nuclear staining (DAPI); green: NLRP3 staining. *n* = 3. * p < 0.05, ** p < 0.01, *** p < 0.001 vs. control. The data are represented as the mean ± SEM. HG, high glucose.

To determine whether high glucose (HG) could induce pyroptosis and serve as an in vitro model for mimicking hyperglycemia and DCM, H9C2 cells were incubated with 30 mM and 50 mM glucose for 24 h, consistent with our previous approach [7]. The results consistently showed a significant dose-dependent increase in the expression of pyroptotic genes, with 50 mM glucose showing a higher induction of pyroptosis. Specifically, NLRP3, GSDMD-N, caspase-1, ASC, IL-18, and IL-1β levels were all significantly increased (Figure 4A,B). Immunofluorescence staining using 4,6-diamidino-2-phenylindole (DAPI) demonstrated that high glucose promoted fragmentation and possibly cell death through pyroptosis (Figure 4C). These results indicate that high glucose triggers the release of pro-inflammatory cytokines, thus generating inflammasomes that promote pyroptosis.

### 2.4. AED Improves the Relative Gene Expression of Pyroptosis in High-Glucose-Exposed H9C2 Cells

**Figure 5:**
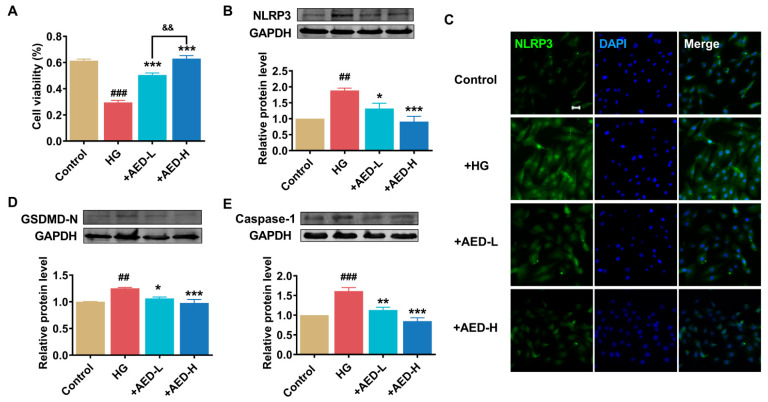
Effects of AED on the production of inflammatory factors in H9C2 cells treated with high glucose. (**A**) Cultured H9C2 cells were pretreated with high glucose (50 mM) for 24 h, followed by AED 10 µM or 20 µM treatment for another 24 h. Protective effects of AED against high-glucose-induced toxicity were investigated using CCK-8 assay. *n* = 3. (**B**) Relative protein levels of NLRP3. *n* = 4. (**C**) Immunofluorescence images (×200) showing the expression of NLRP3 in H9C2 cells. Scale bar: 20 μm. Blue: nuclear staining (DAPI); green: NLRP3 staining. *n* = 3. (**D**,**E**) Relative protein expression levels of GSDMD-N and caspase-1. *n* = 4. * *p* < 0.05, ** *p* < 0.01, *** *p* < 0.001 vs. HG; ^##^ *p* < 0.01, ^###^ *p* < 0.001 vs. control; ^&&^ *p* < 0.01 vs. +AED-L. The data are represented as means ± SEM. HG, high glucose; AED-L, aloe-emodin derivative low concentration; AED-H, aloe-emodin derivative high concentration.

The results consistently demonstrate that high glucose could significantly reduce cell viability. The low concentration of AED (10 µM) and the high concentration of AED (20 µM) were determined based on their ability to enhance cell viability. Notably, a significant dose-dependent increase in cell viability was observed in the AED-treated group compared to high glucose alone (Figure 5A). Subsequently, the effects of AED on the expression of different markers involved in pyroptosis were studied. Immunofluorescence analysis indicated that high-glucose stimulation promoted DNA fragmentation and pyroptotic cell death in H9C2 cells (Figure 5C). The protein levels of NLRP3, GSDMD-N, and caspase-1 were significantly increased in the high-glucose group (Figure 5B,D,E). In contrast, treatment with AED resulted in a significant reduction in the expression levels of these markers. Furthermore, the optimum concentration of AED 20 µM displayed higher potency, as manifested by the remarkable decrease in pyroptosis-associated protein markers.

### 2.5. AED Alleviates Pyroptosis Possibly via Inhibition of NLRP3

**Figure 6:**
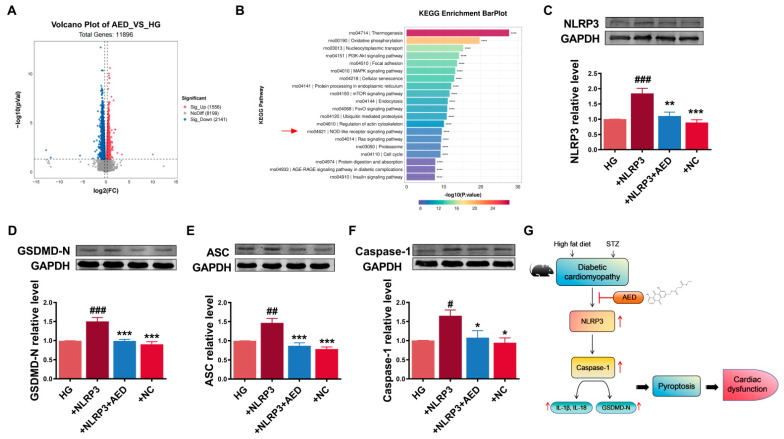
Overexpression of NLRP3 triggers pyroptosis. (**A**) The volcano map of RNA sequencing data from AED 20 µM pretreated with high glucose compared to the high-glucose group. Upregulated genes are marked in light red; downregulated genes are marked in light blue. (**B**) KEGG pathway analysis. **** *p* < 0.0001 (**C**–**F**) Relative protein expression levels of NLRP3, GSDMD-N, ASC, and caspase-1 in H9C2 cells. *n* = 4–5. * *p* < 0.05, ** *p* < 0.01, *** *p* < 0.001 vs. +NLRP3 group; ^#^ *p* < 0.05, ^##^ *p* < 0.01, ^###^ *p* < 0.001 vs. HG group. The data are represented as the mean ± SEM. HG, high glucose; AED, aloe-emodin derivative; NC, negative control. (**G**) Schematic depicting a mechanism of AED in DCM.

In order to determine the potential therapeutic target of AED in H9C2 cell lines under high-glucose conditions, differentially expressed genes (DEGs) analysis was conducted using RNA sequencing data. As shown in Figure 6A, there were a total of 1556 significantly upregulated and 2141 significantly downregulated genes in the AED treatment group compared to the high-glucose group. The Kyoto Encyclopedia of Genes and Genomes (KEGG) pathway analysis of DEGs showed significantly enrichment in the NOD-like receptor signaling pathway (Figure 6B). It was known that NLRP3 is unique among the NOD-like family of receptors in that it is the only known member to be activated, seemingly indirectly, by numerous pathogenic and sterile inflammatory signals [23]. The activation of the NLRP3 inflammasome triggers a series of downstream reactions that culminate in the release of inflammatory cytokines, eventually leading to pyroptosis and cell death [24]. To investigate whether NLRP3 acts as a downstream target of AED, NLRP3-encoded plasmid was used to overexpress the NLRP3 levels in H9C2 cells. The effects of NLRP3 revealed a remarkable increase in the levels of these pyroptosis markers, whereas AED treatment has inhibitory effects on their expression (Figure 6C–F). 

## 3. Discussion

In our present study, we discovered that both AE and AED have the ability to significantly improve cardiac function in DCM. Additionally, our findings indicate that AED exhibits a greater potency compared to AE. An interesting finding from our study is that AED primarily exerts its effects by inhibiting myocardial pyroptosis (Figure 6G). This novel mechanistic insight adds to our understanding of the therapeutic potential of AED in treating DCM.

The development of DCM has been suggested to be multifactorial. Putative mechanisms include: (1) Metabolic abnormalities, such as hyperglycemia, dyslipidemia, and insulin resistance, are all implicated in the pathophysiology of DCM. Hyperglycemia, in particular, has been identified as the main trigger of cardiomyopathy [4,25,26]; (2) Oxidative stress causing deleterious alterations in the diabetic heart resulted into cardiac dysfunction [27]; (3) Mitochondrial dysfunction resulting from alteration of mitochondrial biogenesis and dynamics can contribute to cardiomyopathy [28]; (4) Calcium dynamics at the subcellular level has recently been linked to hypertrophic cardiomyopathy in adult cardiomyocytes [29]; (5) Inflammatory responses resulting from various stimuli have been shown to cause cardiac fibrosis and cardiomyocyte pyroptosis, ultimately leading to cardiac dysfunction [30].

Recently, pyroptosis has gained tremendous attention in the study of cardiac diseases in an attempt to unfold the mystery behind its association with heart diseases and to understand the markers and pathways involved in the processes. Both in vitro and in vivo reports have implicated pyroptosis as an important player in cardiac cell death and fibrosis, eventually leading to DCM [6,31]. More recent studies have shown that inflammasomes associating DCM have emerged as important mediators and regulators of cell death. Activation of NLRP3 inflammasome has been overwhelmingly reported as a DCM marker and is considered to be one of the key molecules involved in triggering the downstream genes leading to cell death via inflammation [32,33]. DCM is characterized by a range of structural and functional disorders, including myocardial fibrosis, left-ventricular dysfunction, cardiomyocyte cell death, and metabolic abnormalities [34]. The death of cardiomyocyte has been premised to be fundamental in triggering cardiac hypertrophy and remodeling, which results in cardiac dysfunction [35]. In this study, we successfully established a type 2 diabetic model in SD rats that manifested myocardial fibrosis, cardiomyocyte cell death, metabolic disorders, and fundamentally cardiac dysfunction. Additionally, the in vitro experiments using H9C2 cell lines displayed elevated levels of NLRP3, ASC, GSDMD-N, caspase-1, IL-18, and IL-1β after exposing to high glucose. These results suggested that the animals treated with a high-fat diet and STZ infection to induce hyperglycemia, as well as H9C2 cells treated with high glucose to simulate glucotoxicity, exhibited signs of pyroptosis. Building upon this observation, we proceeded to determine whether AED could alleviate these abnormalities to maintain a stable cardiac function and to elucidate the possible pathway involved in this process. 

The clinical application of AE has been limited by multiple adverse effects, poor intestinal absorption, short elimination half-life, and low bioavailability in vivo [36]. The substitution of the functional group in anthraquinones may play a crucial role in the anti-inflammatory effectiveness of AED [22]. Specifically, AE has a hydroxymethyl group substitution at the third position, whereas AED has a diethyl succinate substitution at the same position. A comparison of the structural characteristics of these two anthraquinones reveals that converting a polar or hydrophilic hydroxymethyl group into a lipophilic succinic group at the third position confers greater pharmacological potency on the modified anthraquinone. The increase in potency can most likely be attributed to the increased lipid solubility and the improved bioavailability observed in vivo, as well as the enhanced uptake by cells in vitro. As mentioned earlier, there are a few documentations on the anti-inflammatory activity of AE. However, the evidence remains insufficient and somewhat inconclusive. Atherosclerosis is a high lethal inflammatory disease associated with endothelial dysfunction. Previous researches conducted by our research group discovered that AED produced an anti-atherosclerosis effect by reinforcing beclin1-regulated autophagy 1 (AMBRA1)-mediated endothelial autophagy [21]. As far as our study is concerned, no studies have documented the anti-pyroptotic activity of AED in DCM. Therefore, our study aimed to explore the beneficial effects of AED in DCM through its anti-pyroptotic properties. Our investigation revealed that AED effectively suppressed the production of inflammatory mediators. In addition, there were remarkable improvements in the morphological structure and cardiac function observed in the animal model, further supporting the role of AED as a potent anti-inflammatory agent derived from aloe plant.

Pyroptosis is characterized by the formation of pores in the cell membrane, swelling of the cytoplasm, rupture of the cell membrane, and eventually the release of cytokines such as IL-1β and IL-18 into the extracellular environment, ultimately leading to cellular death [37]. When triggered, the inflammasome activation of the NLRP3 oligomerization and ASC recruitment domains triggers the auto-cleaving of pro-caspase into caspase-1. The active caspase-1 then promotes the maturation of IL-1β and IL-18 and cleaves GSDMD to form GSDMD-N and pore formation on the cell membrane through which the cytokines are secreted, inducing pyroptosis [38,39]. In our current study, we observed a remarkable decline in the connection between the expression of NLRP3 and the secretion of interleukins in pyroptotic cells in vivo. This was manifested by less injury, fewer structural changes, and reduced cell death in the diabetic drug-treated groups compared to the DCM group. These findings demonstrate that the activation of NLRP3 and subsequent pyroptosis play a pivotal role in the development of DCM, and that this dysfunction could be corrected by AED. Additionally, we proved that the NLRP3 protein level was significantly upregulated in NLRP3-plamid-transfected cells. This finding aligns with previous reports that NLRP3 activation and overexpression contributed to DCM through pyroptosis-mediated processes [40]. Furthermore, AED exhibited notable anti-inflammatory activity by downregulating the protein levels.

To the best of our knowledge, our study represents the first investigation demonstrating the ability of AED to mitigate pyroptosis in DCM, both in vitro and in vivo. Targeting the NLRP3 inflammasome and pyroptosis may offer a novel strategy to better understand the treatment of DCM. However, this study has several limitations that require further research. Firstly, our investigation focused solely on the impact of NLRP3 overexpression as an enhancer of inflammation and pyroptosis. Further studies should explore the effects and the roles of other specific inflammatory downstream genes involved in DCM. Additionally, it would be valuable to verify the specific effects of the NRLP3 inflammasome when NLRP3 is knocked-out or silenced in DCM rats. This can also help to further evaluate the upstream signaling pathways involved in NLRP3-inflammasome-induced pyroptosis in DCM. The development of therapeutic compounds that specifically target the activation of NLRP3 inflammasome and pyroptosis holds promise for the mitigation of cardiovascular diseases induced by pyroptosis. 

## 4. Materials and Methods

### 4.1. Animals and Establishment of DCM Model

SD rats (180–220 g) were housed at an ambient temperature of 22 ± 1° C and maintained at humidity levels of 55 ± 5%, with food and water available ad libitum. After acclimation for a week, the rats were randomly divided into 6 groups (*n* = 8): control, DCM, AED low concentration (AED-L, 50 mg/kg/day), AED high concentration (AED-H, 100 mg/kg/day), AE (100 mg/kg/day), and positive control (Glyburide, 2 mg/kg/day [41]) groups. AE (Cat. no. MB5996) and glyburide (Cat. no. MB1563) were purchased from Dalian Meilun Biotech Co., Ltd. (Dalian, China). AED, obtained from the Department of Pharmaceutical Chemistry, Harbin Medical University, was synthesized via AE reacted with monoethyl succinate. Drugs were dissolved in 0.5% carboxy methyl cellulose (CMC) (Solarbio, Beijing, China). The rats in the control group were fed with a basal diet, while the rest of the animals were fed with a high-fat diet (34.5% fat, 17.5% protein, and 48% carbohydrate) for 4 weeks. The high-fat-diet rats were then intraperitoneally injected with STZ 30 mg/kg (Sigma, Saint Louis, MO, USA) dissolved in 0.1 M citrate buffer solution (pH = 4.3) for 3 days [42]. The plasma glucose levels were measured in fasting condition. The rats that attained fasting blood glucose of ˃16.7 mmol/L were considered successful models. Different drugs were intragastrically administered for 4 weeks. Rats in the control and DCM groups were treated with the same volume of CMC intragastrically. A total of 3–5 mL of the solution was administered according to the body weight on a daily basis. Rats were anesthetized by intraperitoneal injection of sodium pentobarbital 200 mg/kg (Sigma) and then sacrificed for tissue harvest.

### 4.2. Echocardiographic Measurements

To further confirm the DCM status and evaluate the efficacy of AED pretreatment, transthoracic echocardiography was performed using an ultrasound machine (Vivid 7, GE Medical, Milwaukee, WI, USA) equipped with a 10 MHz phased array transducer. Adult rats were anesthetized by intraperitoneal injection of sodium pentobarbital (40 mg/kg). Left-ventricular internal dimensions (LVID; s) (LVID; d), and interventricular septum thickness (IVS; s and IVS; d) were each measured during systole and diastole. M-mode tracings were used to determine the percentage of ejection fraction and fractional shortening.

### 4.3. Fasting Blood Glucose

Blood glucose collection from the tail of the rats that fasted overnight was performed using sterile lancets to puncture the skin and to collect a drop of blood. The glucose test was conducted on a digital glucometer (Accu-Chek, Roche, Indianapolis, IN, USA).

### 4.4. Transmission Electron Microscopy

Tissue specimens were prepared by routine methods [43]. Briefly, the samples were fixed in 2.5% glutaraldehyde at 4 °C in 0.1 mol/L phosphate-buffered saline (PBS) (pH = 7.35) for 24–48 h and afterwards post-fixed in 1% osmium tetroxide for 2 h. The sections were then subjected to 1% uranyl acetate, dehydrated in graded ethanol and embedded in epoxy resin by routine methods. The stained sections were then electron-stained and observed under a JEM-1200 electron microscope (JEOL Ltd., Tokyo, Japan).

### 4.5. H&E and Masson’s Trichrome Staining

The tissues were fixed in 4% paraformaldehyde solution followed by dehydration. The samples were then embedded in paraffin wax, sliced into 5 μm thick sections using microtome and stained with H&E and Masson’s trichrome staining (Solarbio, Beijing, China) to assess the degree of fibrosis, inflammation, and vascular proliferation microscopically.

### 4.6. Cell Culture and Transfection

The H9C2 embryonic rat-heart-derived cell line (American Type Culture Collection, Rockville, MD, USA) was maintained in Dulbecco’s modified Eagle’s medium (DMEM; Gibco, Carlsbad, CA, USA) containing 10% fetal bovine serum (Gibco, Carlsbad, CA, USA) and supplemented with 1% penicillin and streptomycin. The cells were cultured at a density of 5 × 10^4^ cells/cm^2^ and equilibrated with humidified air containing 5% CO_2_ at 37 °C. H9C2 cells were transiently transfected with NLRP3 plasmid. For transient expression experiments, cells were incubated in 6-well plates and used after attaining a confluence of 80%. The transfection mixture (100 nM) was dissolved in Opti-MEM serum-free media (Gibco, Carlsbad, CA, USA) and added to the cells. After 6 h, the medium was replaced by fresh medium with high glucose at 50 mM for 24 h, and then treated with or without AED for another 24 h, after which the cells were used for protein/RNA extraction.

### 4.7. Cell Viability Assay

Cell viability was detected using Cell Counting Kit-8 (CCK-8; Beyotime, Shanghai, China). H9C2 cells were seeded in 96-well plates and incubated with high glucose, followed by treatment with different concentrations of AED for 24 h. CCK-8 reagent (10 µL) was added into each well and incubated for 1 h according to the manufacturer’s instructions. Absorbance measurements were taken at 450 nm on a microplate reader.

### 4.8. RNA Extraction and Quantitative Real-Time PCR

Total RNA was extracted from the rat heart (previously stored in liquid nitrogen) and H9C2 cells using TRIzol LS (Invitrogen, Carlsbad, CA, USA) according to the manufacturer’s instructions. The RNA concentration was detected using a NanoDrop spectrophotometer (NanoDrop Technologies, Wilmington, DE, USA). The RNA was then reverse-transcribed using a reverse-transcription kit (Applied Biosystems, Carlsbad, CA, USA, Cat. no. 4368814) according to the manufacturer’s instructions. The SYBR Green PCR Mix Kit (Applied Biosystems, Cat. no. 4309155) was used to measure the relative mRNA levels of NLRP3, ASC, GSDMD-N, caspase-1, IL-18, and IL-1β. Real-time PCR was conducted with the 7500 FAST RealTime PCR System (Applied Biosystems) for 40 cycles, using the glyceraldehyde 3-phosphate dehydrogenase (GAPDH) gene as a reference housekeeping gene. The sequences of primer pairs used in our study are as follows:

IL-1β: F: 5′-ACTTGGGCTGTCCAGATGAG-3′;
R: 5′-GTAGCTGCCACAGCTTCTCC-3′;
IL-18: F: 5′-GCTCTGGGATGGATTGAAGA-3′;
R: 5′-TCAAGGTCATGCTGTGGTTG-3′;
GAPDH: F: 5′-AGTTCAACGGCACAGTCAAG-3′;
R: 5′-TACTCAGCACCAGCATCACC-3′.

### 4.9. Western Blotting

Protein samples were loaded at 60–100 μg/well into a 10% SDS-PAGE gel for electrophoresis and transferred onto nitrocellulose filter membranes (PALL, New York, NY, USA), which were subsequently blocked in 5% non-fatted milk dissolved in PBS for 2 h. The blots were probed with specific primary antibodies against NLRP3 (1:1000, Cat. no. 15101; Cell Signaling Technology, Danvers, MA, USA), ASC (1:1000, Cat. no. 67824; Cell Signaling Technology, Danvers, MA, USA), GSDMD-N (1:500, Cat. no. 93709; Cell Signaling Technology, Danvers, MA, USA), and caspase-1 (1:500, Cat. no. 22915-1-AP; Proteintech, Chicago, IL, USA) and with GAPDH (1:1000, Cat. no. TA-08; ZSGB-BIO, Beijing, China) as an internal control. The membranes were then incubated on a shaker at 4 °C overnight with the primary antibody. The following day, membranes were incubated with horseradish-peroxidase-conjugated goat anti-mouse IgG or anti-rabbit IgG (1:5000; ZSGB-BIO, Beijing, China) for 1 h. Western blotting bands were quantified using the Odyssey Infrared Imaging System (LI-COR, Lincoln, NE, USA) by measuring band intensity. 

### 4.10. Immunofluorescence Staining and Immunohistochemistry

For immunofluorescence staining, H9C2 cells were cultured on chamber slides and then fixed with 4% buffered paraformaldehyde in PBS. Blocking solution (1% bovine serum albumin and 0.1% Triton-X in PBS) was used to increase permeability and was kept at room temperature for 2 h. Primary antibodies against caspase-1 were placed in PBS overnight at 4 °C. The slides were then incubated with the specific secondary antibody (Invitrogen, Carlsbad, CA, USA) for 1 h at room temperature, followed by nuclei staining using DAPI (Beyotime, Shanghai, China) for 20 min at room temperature. The stained sections were then analyzed with a fluorescence microscope (Nikon 80i, Otawara, Tochigi, Japan). 

For immunohistochemical analysis, frozen heart section specimens obtained from rat tissues were fixed with 4% buffered paraformaldehyde embedded in paraffin. Ascending series of ethanol cleared with xylene were added to the specimens for dehydration. The sections were then immune-stained with primary antibodies against NLRP3 at 4 °C overnight before being washed three times with PBS and incubated with secondary antibody for one hour. The sections were then stained with diaminobenzidine. The images were captured by a fluorescence microscope. 

### 4.11. RNA Extraction Library Construction and RNA Sequencing

Total RNA of H9C2 cells under treatment of high glucose with or without AED 20 µM was isolated using TRIzol reagent (Invitrogen, USA) following the manufacturer’s procedure. The total RNA quantity and purity were analyzed using Bioanalyzer 2100 and RNA 6000 Nano LabChip Kit (Agilent, Santa Clara, CA, USA, 5067-1511), and high-quality RNA samples with RIN number > 7.0 were used to construct the sequencing library. Following purification, the mRNA was fragmented into short fragments. Then, the cleaved RNA fragments were reverse-transcribed to create the cDNA, which were next used to synthesize U-labeled second-stranded DNAs. An A-base was then added to the blunt ends of each strand, preparing them for ligation to the indexed adapters. Each adapter contained a T-base overhang for ligating the adapter to the A-tailed fragmented DNA. Dual-index adapters were ligated to the fragments, and size selection was performed with AMPureXP beads. After the heat-labile UDG enzyme treatment of the U-labeled second-stranded DNAs, the ligated products were amplified with PCR. A cDNA library constructed by technology from the pooled RNA was sequenced run with Illumina NovaseqTM 6000 sequence platform. Using the Illumina paired-end RNA-seq approach, we sequenced the transcriptome, generating a total of million 2 × 150 bp paired-end reads. Reads were further filtered to obtain high-quality clean reads by Cutadapt (v1.9). We aligned reads of all samples to the Rattus norvegicus reference genome using HISAT2 (v2.2.1) package. The mapped reads of each sample were assembled using StringTie (v2.1.6) with default parameters. Then, all transcriptomes from all samples were merged to reconstruct a comprehensive transcriptome using gffcompare software (v0.9.8). After the final transcriptome was generated, StringTie and ballgown were used to estimate the expression levels of all transcripts and perform expression abundance for mRNAs by calculating the fragment per kilobase of transcript per million mapped reads (FPKM) value. 

### 4.12. Analysis of DEGs and KEGG Pathways Enrichment

Gene differential expression analysis was performed by DESeq2 software between two different groups. The genes with the parameter of *p* value < 0.05 and absolute fold change ≥1.2 were considered DEGs. Then, DEGs were subjected to enrichment analysis of KEGG pathways. Pathways meeting this condition with *p* value < 0.05 were defined as significantly enriched pathways. 

### 4.13. Statistical Analysis

All analyses were carried out in GraphPad Prism 9. One-way analysis of variance (ANOVA) and the Tukey–Kramer test were used to compare the difference among groups, and unpaired t-tests were used for differences between two groups. Each experiment was repeated at least 3 times. Continuous variables are presented as mean ± SEM. *p* < 0.05 was regarded as statistically significant.

## 5. Conclusions

In summary, our research demonstrates that hyperglycemia activates the NLRP3 inflammasome signaling pathway, leading to cardiomyocyte pyroptosis. Our findings suggest that AED has the potential to inhibit pyroptosis and attenuate DCM. Targeting the pyroptosis pathway mediated by the NLRP3 inflammasome could provide a novel therapeutic strategy for enhancing our understanding and treatment of DCM. Moreover, AED shows promise as a therapeutic compound to improve cardiac function in DCM. These findings not only broaden and deepen our understanding of the pharmacological properties of derivatives of traditional Chinese medicine monomers, but also highlight their potential as novel therapeutics for DCM. 

## Data Availability

Data is contained within the article.
